# Transmembrane Transport of Bicarbonate by Anion Receptors

**DOI:** 10.1002/cplu.202200266

**Published:** 2022-11-22

**Authors:** Luis Martínez‐Crespo, Hennie Valkenier

**Affiliations:** ^1^ Department of Chemistry University of Manchester Oxford Road Manchester M13 9PL UK; ^2^ Manchester Institute of Biotechnology University of Manchester 131 Princess Street Manchester M1 7DN UK; ^3^ Université Libre de Bruxelles (ULB) Engineering of Molecular NanoSystems Ecole polytechnique de Bruxelles Avenue F.D. Roosevelt 50, CP165/64 B-1050 Brussels Belgium

**Keywords:** anion receptors, bicarbonate, ion transport, membranes, supramolecular chemistry

## Abstract

The development of synthetic anion transporters is motivated by their potential application as treatment for diseases that originate from deficient anion transport by natural proteins. Transport of bicarbonate is important for crucial biological functions such as respiration and digestion. Despite this biological relevance, bicarbonate transport has not been as widely studied as chloride transport. Herein we present an overview of the synthetic receptors that have been studied as bicarbonate transporters, together with the different assays used to perform transport studies in large unilamellar vesicles. We highlight the most active transporters and comment on the nature of the functional groups present in active and inactive compounds. We also address recent mechanistic studies that have revealed different processes that can lead to net transport of bicarbonate, as well as studies reported in cells and tissues, and comment on the key challenges for the further development of bicarbonate transporters.

## Graphical abstract

**The development of synthetic compounds** for the transmembrane transport of bicarbonate is discussed in this Review, presenting the structures of the different transporters and the methods to study the transport process in liposomal membranes, together with the most recent studies on transport mechanism and biological activity in cells and tissues.

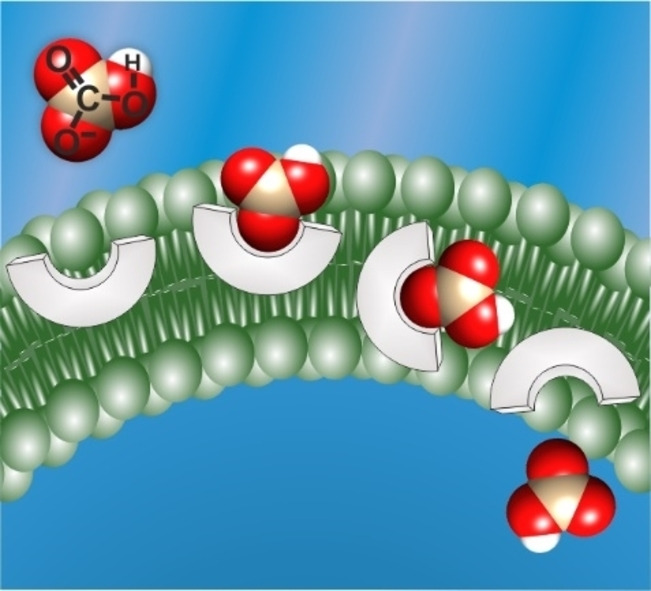

## 1 Introduction

**Scheme 1:**
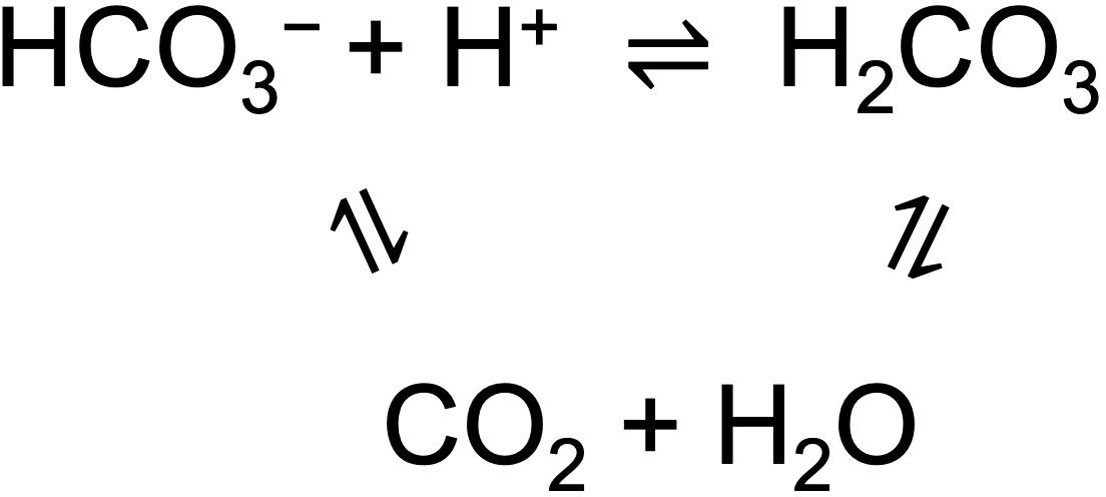
Overview of the interconversion equilibria between bicarbonate, carbonic acid, and carbon dioxide.

Bicarbonate is, after chloride, the most abundant anion in extracellular fluids, with concentrations of 22–26 mM in blood serum and cerebrospinal fluid.^[1]^ Key purposes of bicarbonate are pH control^[2]^ and transport of metabolic waste. Both these functions are based on the equilibria between bicarbonate anions and its related neutral species (Scheme 1). HCO_3_^−^ is the conjugate base of H_2_CO_3_, which has a pK_a_ of 3.5.^[3]^ However, H_2_CO_3_ is rapidly dehydrated to form CO_2_, and CO_2_ plus H_2_O can also interconvert into HCO_3_^−^ and H^+^ directly.^[4]^ The existence of these equilibria results in an apparent pK_a_ of 6.35 for H_2_CO_3_ in presence of dissolved CO_2_.^[3]^ This apparent pK_a_ value, combined with the pK_a_ of 10.3 for the HCO_3_^−^/CO_3_^2−^ equilibrium, makes bicarbonate an important buffer in biology. As the concentration of CO_2_ in aqueous solutions is three orders of magnitude higher than that of H_2_CO_3_,^[5]^ the presence of carbonic acid can generally be ignored.

We note that ‘bicarbonate’ is a trivial name that originates from the carbonate to calcium stoichiometry in Ca(HCO_3_)_2_ and that the IUPAC recommends the use of ‘hydrogencarbonate’, which better describes the HCO_3_^−^ anion.^[6]^ Since the literature on anion transporters that we surveyed uses the trivial name ‘bicarbonate’, we will use this name in the current review.

### 1.1 Bicarbonate transport in biological systems

The transport of HCO_3_^−^ across lipid bilayer membranes plays an important role in many different organs and tissues.^[7]^ In contrast to neutral CO_2_, which can diffuse spontaneously across the membranes of various cells and organelles,^[8]^ the transport of anionic HCO_3_^−^ requires membrane proteins. Bicarbonate transport proteins can perform electroneutral Cl^−^/HCO_3_^−^ exchange,^[9]^ exchange of HCO_3_^−^ or Cl^−^ with other anions in various stoichiometries,^[10]^ or Na^+^‐coupled HCO_3_^−^ transport.^[11]^ In addition, certain anion channels, including the Cystic Fibrosis Transmembrane conductance Regulator (CFTR), can also transport HCO_3_^−^.^[12]^

**Figure 1:**
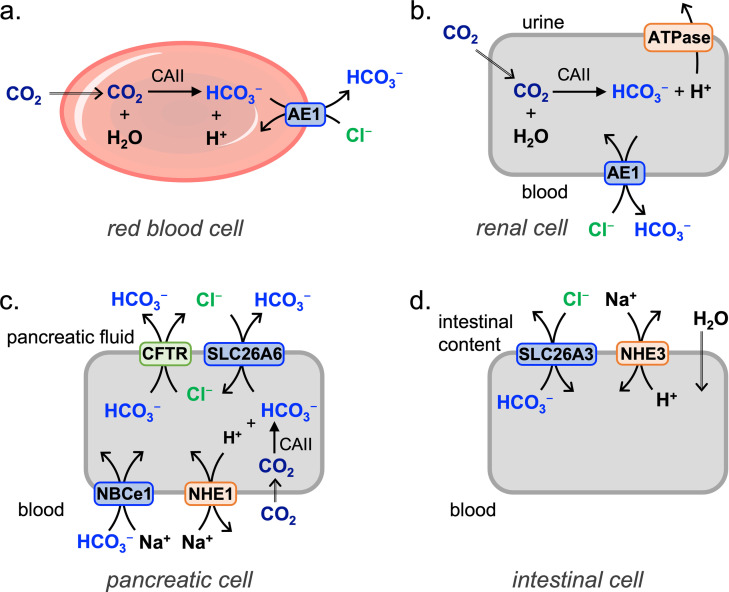
Simplified schemes showing examples of bicarbonate transport in different cells: a. red blood cells that convert CO_2_ into HCO_3_^−^ to facilitate transport of metabolic waste via the blood stream to the lungs, where the reverse process takes place, b. renal cells that ensure acidification of urine and reabsorption of HCO_3_^−^ into blood, c. pancreatic cells that excrete HCO_3_^−^ into the pancreatic fluid, and d. intestinal cells that absorb NaCl and H_2_O from the intestinal content into the blood stream.

A first example of bicarbonate transport is found in red blood cells, which take up CO_2_ that is excreted from cells as a waste product of aerobic respiration. In the red blood cells, CO_2_ is converted into HCO_3_^−^ and H^+^ by carbonic anhydrase II (CAII). The HCO_3_^−^ transporter AE1 then transports HCO_3_^−^ out of the red blood cells, in exchange for Cl^−^ (Figure 1a). This process prevents acidification of tissues, while the resulting acidification of the red blood cells enhances the O_2_ release by haemoglobin. The released HCO_3_^−^ is more soluble in the blood plasma than CO_2_ is, allowing the HCO_3_^−^ to travel to the lungs via the blood stream. In the lungs, the reverse processes will take place: HCO_3_^−^ is transported into red blood cells (and Cl^−^ out) by AE1, after which the CO_2_ diffuses out of the red blood cells to get exhaled.^[13]^

A second example is found in kidneys, where reabsorption of HCO_3_^−^ from the urine into the blood takes place to avoid acidification of the organism. CO_2_ diffuses from the urine into renal cells to get converted into HCO_3_^−^ and H^+^ by CAII, resembling the process in red blood cells. The H^+^ is transported back into the urine by ATPase to give a net absorption of HCO_3_^−^. The HCO_3_^−^ is then transported into the blood by the HCO_3_^−^/Cl^−^ exchanger AE1 in certain renal cells (Figure 1b), while in others the protein NBCe1 performs symport of HCO_3_^−^ and Na^+^.^[13]^

The gastrointestinal tract provides further examples. In certain gastric cells, HCO_3_^−^/Cl^−^ transporters play an important role in attaining the concentrations of HCl inside the cell as required for the excretion of gastric acid. Subsequently, the pancreas excretes pancreatic fluid with HCO_3_^−^ concentrations as high as 140 mM to neutralise the gastric acid in the first part of the small intestines.[^7^, ^14^] Pancreatic cells take HCO_3_^−^ from the blood to transfer it to the pancreatic fluid. This is achieved by a combination of HCO_3_^−^/Cl^−^ exchange by proteins from the SLC26A family and HCO_3_^−^ transport by the CFTR (Figure 1c), in a similar way as these proteins team up in the lungs to secrete Cl^−^ and HCO_3_^−^ into the airway surface liquid (see Section 4.3).[^7^, ^15^] Furthermore, in the intestines the HCO_3_^−^/Cl^−^ exchange by transport protein SLC26A3 is coupled to H^+^/Na^+^ exchange by NHE3 (Figure 1d). This leads to the net reabsorption of NaCl, which in turn drives the reabsorption of water.

These are only a few examples of the numerous crucial biological processes that involve transmembrane transport of HCO_3_^−^ in humans and animals.[^7^, ^14^] The importance of HCO_3_^−^ transport is further demonstrated by diseases that are linked to mutations in HCO_3_^−^ transporting proteins. Examples are renal tubular acidosis, congenital chloride diarrhoea, Pendred syndrome, glaucoma, and various blood disorders.[^7^, ^10^, ^11^, ^14^] Altered expression patterns of HCO_3_^−^ transporters have been observed in many types of cancer as well.^[16]^ Many symptoms of the congenital disease *cystic fibrosis* (CF), primarily associated with deficient Cl^−^ transport by CFTR, are also caused by a lack of HCO_3_^−^ crossing epithelial cell membranes.[^12^, ^17^, ^18^]

Little evidence for transmembrane transport of HCO_3_^−^ has been found in terrestrial plants, that readily take up CO_2_ for photosynthesis from the air.^[19]^ In contrast, HCO_3_^−^ transport is of great importance for aquatic organisms, such as algae,^[20]^ cyanobacteria,^[21]^ and seagrasses.^[22]^ Firstly, HCO_3_^−^ concentrations are much higher than CO_2_ concentrations in most aquatic environments. Secondly, HCO_3_^−^ transport is essential for the CO_2_ concentration mechanism (CCM) in these organisms. Transport of HCO_3_^−^ into the cells and organelles and subsequent conversion into CO_2_ by carbonic anhydrase results in elevated CO_2_ concentrations close to the enzyme Rubisco, which is responsible for CO_2_ fixation in the Calvin cycle.^[23]^

Binding sites for bicarbonate in proteins are not always clearly defined and vary from structure to structure. However, hydrogen bonding is the main interaction and water molecules or cations (such as Na^+^ and Ca^2+^) have also been found close to the anion.[^24^, ^25^]

### 1.2 Development of synthetic bicarbonate transporters

**Figure 2:**
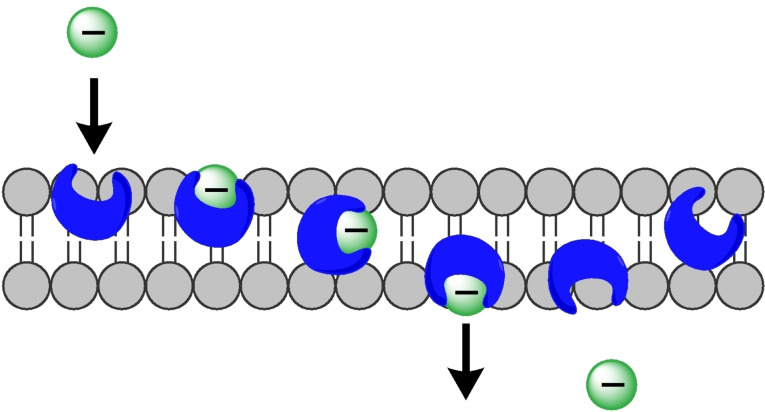
Schematic representation of anion transport across a membrane by a synthetic receptor acting as a mobile carrier.

Supramolecular chemists have envisaged to perform the transport of HCO_3_^−^ using synthetic anion receptors.[^26^, ^27^, ^28^] These compounds can bind the HCO_3_^−^ anion and move across lipid membranes as mobile carriers, releasing the anion on the other side (Figure 2). Such transporters are also referred to as anionophores. The development of HCO_3_^−^ transporters was defined as “an interesting target with potential utility” by A. P. Davis, Sheppard, and Smith in 2006,^[29]^ who had first investigated Cl^−^/HCO_3_^−^ transport by a cholapod in 2003.^[30]^ After this initial study, J. T. Davis, Gale, Quesada and co‐workers published a seminal article on HCO_3_^−^ transporters in 2009, in which they reported a series of isophthalamides and the natural compound prodigiosin to act as Cl^−^/HCO_3_^−^ exchangers.^[31]^ Since then, many different anion transporters have been shown to transport HCO_3_^−^, which will be discussed in the Section 3 of this review.

The development of synthetic HCO_3_^−^ transporters is primarily motivated by the perspective of applications in medicine, due to the great importance of HCO_3_^−^ transport in humans and the various diseases linked to impaired HCO_3_^−^ transport. Secondly, considering the importance of HCO_3_^−^ transport in the context of CO_2_ concentration in algae and cyanobacteria, synthetic HCO_3_^−^ transporters are also interesting tools to study this mechanism towards the engineering of CCM in plants,^[23]^ or artificial CO_2_ fixation.^[32]^ Thirdly, HCO_3_^−^ transporters could find applications in sensing, as components of ion selective membranes.[^33^, ^34^] While ion selective electrodes (ISE) are commercially available for different anions and cations, the development of an ISE for HCO_3_^−^ has proven to be challenging, due to the different equilibria in which this anion is involved and the lack of selective anionophores for HCO_3_^−^.[^35^, ^36^, ^37^, ^38^]

## 2 Methods to study bicarbonate transport using vesicles

The development of transmembrane anion transporters requires well‐established methods that permit to assess the transport properties of the compounds to study. Cl^−^ can be directly detected either with an ion selective electrode (ISE) or with fluorescent probes such as lucigenin or SPQ, and such detection systems can be used to develop transport assays using large unilamellar vesicles (LUVs, also referred to as liposomes) as a model of the cell membrane. On the other hand, similar detection systems for HCO_3_^−^ have only been available recently. Consequently, the ability of anionophores to transport HCO_3_^−^ has mainly been studied by indirect methods that monitor Cl^−^ transport. Such methods assume that the transport of Cl^−^ in one direction is compensated by the transport of HCO_3_^−^ in the opposite direction to maintain the electroneutrality of the intra‐ and extravesicular media, giving a global process called Cl^−^/HCO_3_^−^ antiport.

**Figure 3:**
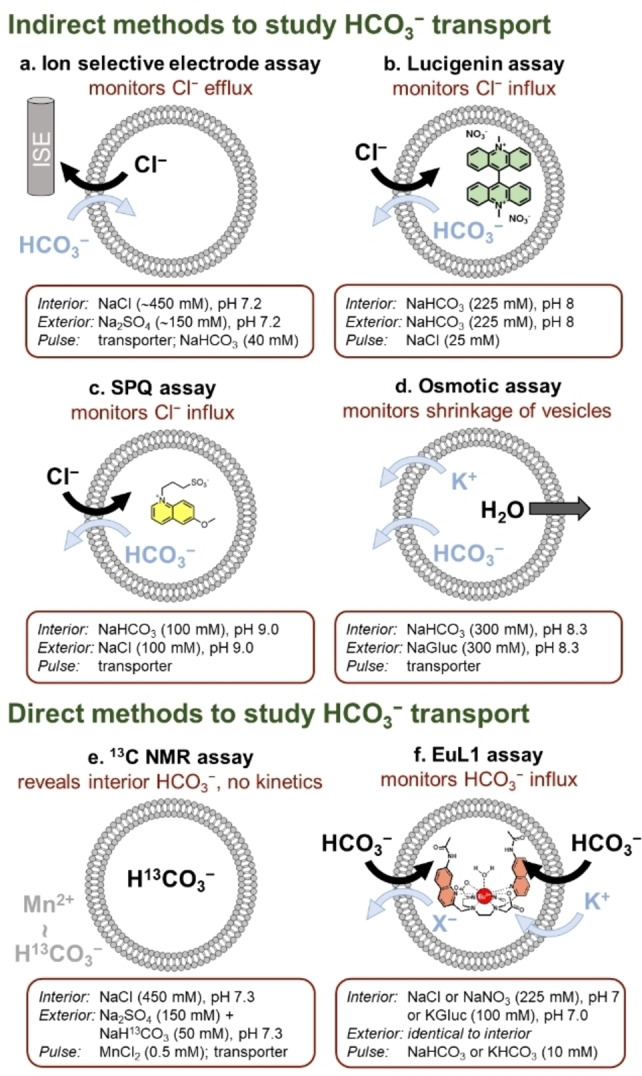
Methods used to study transmembrane transport of HCO_3_^−^ in large unilamellar vesicles (LUVs). The black arrows indicate the transport processes that are monitored.

The chloride selective electrode or ion selective electrode (ISE) assay was first used to study HCO_3_^−^ transport in 2003 by Smith, A. P. Davis and co‐workers^[30]^ and then optimized and largely exploited in the labs of Gale and Quesada.^[31]^ In this assay, the vesicles are charged with NaCl and suspended in Na_2_SO_4_, and the efflux of Cl^−^ is monitored with an ISE that reports on the extravesicular concentration of this anion. Upon addition of the transporter, dissolved in an organic solvent, no significant response is observed because SO_4_^2−^ is too hydrophilic to be transported. Then, the transport process is triggered by an extravesicular pulse of NaHCO_3_ and monitored for ∼300 s (Figure 3a). The concentration of transporter required to obtain efficient transport will depend on the activity of the transporter. Normally, transport curves for different concentrations of a transporter are recorded and the levels of chloride released at an established time are used to construct a dose‐response curve. Then this curve is fitted to the Hill equation to obtain an EC_50_ value, which corresponds to the concentration of transporter required to obtain 50 % of the maximum response. The EC_50_ value is thus indicative of the activity of the transporter.

Lucigenin was first used to study Cl^−^/HCO_3_^−^ antiport in 2010 by the group of J. T. Davis,^[39]^ and it has also been used by the groups of A. P. Davis, Valkenier, Schmitzer, and Liu. In the most common lucigenin assay, the vesicles are prepared with lucigenin inside and NaHCO_3_ inside and outside. The transporters can be added in an organic solvent to the vesicles after their formation (postinsertion), as in the ISE assay, or during the preparation of the vesicles (preincorporation). The transport starts with an extravesicular pulse of NaCl and is monitored by fluorescence spectroscopy, showing the quenching of lucigenin caused by the Cl^−^ transported into the vesicles (Figure 3b).^[40]^ The transport curves obtained will reflect the activity of a transporter at a concrete concentration. In some cases, the transport activity has been quantified by fitting the curves using first and second order exponential decay functions to obtain initial rate and half‐life values, respectively.^[41]^ While the initial rate corresponds to the rate of transport at the beginning of the process, the half‐life is the time required to reach half of the total response, and both reflect the efficiency of the transport. Moreover, the specific initial rate [*I*] can be calculated to obtain a value which is independent of the concentration of transporter, which facilitates the comparison between different transporters. In some labs, the lucigenin is encapsulated in the vesicles with a solution of NaCl and the vesicles are dispersed in a solution of NaHCO_3_. Under these conditions the transport process is triggered by the addition of the transporter and only postinsertion is possible. In this case, the transport is monitored as the increase in fluorescence intensity caused by the release of chloride from the vesicles.

Ko, Yao, Dan Yang and co‐workers have studied Cl^−^/HCO_3_^−^ antiport using 6‐methoxy‐N‐(3‐sulfopropyl) quinolinium (SPQ), which has similar properties to those of lucigenin.^[42]^ With vesicles charged with SPQ and NaHCO_3_ and suspended in NaCl, the transport initiated by addition of a transporter can be monitored by following the quenching of the dye (Figure 3c).

In their pioneering work, J. T. Davis, Gale, Quesada and co‐workers developed an assay based on ^13^C NMR to obtain direct evidence of transmembrane transport of bicarbonate.^[31]^ This assay has been used in various studies to complement the results of some of the methods described above. This assay uses NMR spectroscopy to detect ^13^C labelled bicarbonate present inside LUVs and the experiments can be set up to study transport of bicarbonate either into (influx) or out of (efflux) the vesicles (Figure 3e). In a typical influx experiment, the vesicles are charged with NaCl and suspended in a medium containing Na_2_SO_4_, NaH^13^CO_3_ and MnSO_4_. The ^13^C NMR spectrum is recorded before and after addition of the transporter. The paramagnetic Mn^2+^ causes the broadening of the signal of extravesicular H^13^CO_3_^−^. Therefore, only a broad signal is observed at the beginning of the experiment, and a sharp signal appears after the addition of transporter, which indicates that H^13^CO_3_^−^ has been transported into the vesicles (probably balanced by Cl^−^ transport in the opposite direction). The same principle is used for the efflux experiment, which uses vesicles initially charged with NaH^13^CO_3_ and suspended in a mixture of Na_2_SO_4_ and NaCl. In this case MnSO_4_ is added after the transporter and the absence of a sharp signal indicates that the H^13^CO_3_^−^ initially encapsulated has been transported out of the vesicles. The main limitation of this assay is that accurate kinetic data of the transport process cannot be obtained, unless working with an NMR spectrometer equipped with a rapid injection system.^[43]^

A more recent strategy to monitor transport of bicarbonate is the osmotic assay developed by Gale and co‐workers.^[44]^ In this method, the vesicles are charged with KHCO_3_ and suspended in K‐Gluconate, and valinomycin, a cation transporter, is added to the vesicles. Then, the addition of an anion transporter triggers the transport of HCO_3_^−^ out of the vesicles, a process that is coupled to the transport of K^+^ by valinomycin in the same direction, resulting in global K^+^/HCO_3_^−^ symport. The decrease in osmotic pressure inside the vesicles will cause the efflux of water and in consequence a decrease of the size of the vesicles (Figure 3d). This shrinking of the vesicles leads to an increase in the amount of scattered light (600 nm), which can be monitored with a fluorescence spectrometer. This assay has the additional interest that it permits to study the ability of the transporters to perform HCO_3_^−^ in one direction, a process known as HCO_3_^−^ uniport.

To further develop HCO_3_^−^ transporters for the different potential applications presented in the previous section, direct and sensitive methods to study and fully understand the transport of this anion are required. Last year, in collaboration with S. Butler, we reported the EuL1 assay, that uses a luminescent europium‐based complex as HCO_3_^−^ probe for the direct monitoring of bicarbonate transport using fluorescence spectroscopy.^[45]^ In this assay, the probe EuL1 is encapsulated inside LUVs which contain NaX (X=Cl or NO_3_) both inside and outside the vesicles. The transporter is added to the membranes by either preincorporation or postinsertion and the transport is triggered by an extravesicular pulse of NaHCO_3_ (Figure 3f). The transport of HCO_3_^−^ into the vesicles is reported by an increase on the emission intensity of the probe and is compensated by the transport of X^−^ in the opposite direction. Furthermore, the EuL1 assay can also be performed with K‐gluconate as the salt inside and outside the vesicles. Under these conditions and in presence of both valinomycin and an anionophore, an extravesicular pulse of KHCO_3_ initiates a process where the transport of HCO_3_^−^ by the anionophore is coupled to transport of K^+^ by valinomycin (Figure 3f). As in the lucigenin assay, the initial rate and half‐life (or rate constant) values can be obtained by fitting the data using second and first order exponential decay functions, respectively. Thus, the EuL1 assay can be used to study HCO_3_^−^/Cl^−^ and HCO_3_^−^/NO_3_^−^ antiport as well as HCO_3_^−^ uniport by direct monitoring of the intravesicular concentration of HCO_3_^−^.

Radiometric assays have been used to monitor transport of H^14^CO_3_^−^ into cells,^[43]^ and to monitor transport of other species, such as the amino acid proline, into LUVs.^[46]^ However, to the best of our knowledge, this methodology has not yet been used to study HCO_3_^−^ transport by synthetic compounds in vesicles.

All the assays described above are generally performed with LUVs formed from phosphatidylcholine‐based lipids. The ISE, ^13^C NMR, and osmotic assays have typically used 1‐palmitoyl‐2‐oleoylphosphatidylcholine (POPC) and egg yolk L‐α‐phosphatidylcholine (EYPC) has been used in the SPQ assay and in the less common variation of the lucigenin assay that encapsulates Cl^−^ to study its release. On the other hand, the common lucigenin and EuL1 assays have generally used a mixture of POPC and cholesterol in a 7 : 3 ratio. Cholesterol decreases membrane fluidity and can thus reduce the rate of diffusion of species embedded in the membrane, such as a transporter‐anion complex, but also of lucigenin.

## 3 Overview of synthetic bicarbonate transporters

### 3.1 Early bicarbonate transporters

**Figure 4:**
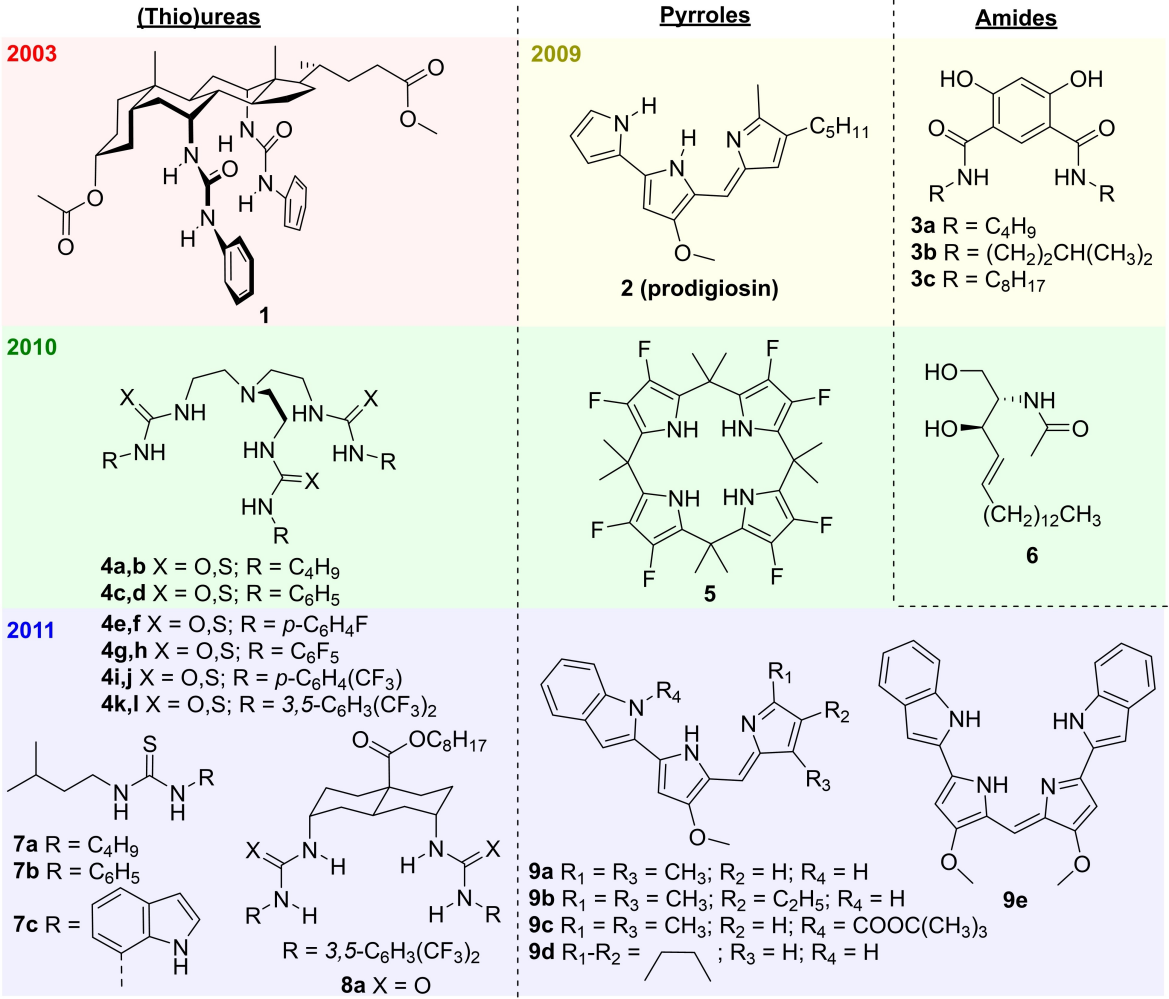
Early examples of small molecules studied as HCO_3_^−^ transporters.

In the first report of an artificial bicarbonate transporter, A. P. Davis and co‐workers studied the chloride transport properties of a family of steroid‐based bis‐ureas named cholapods.^[30]^ To study the transport of those compounds, the authors performed ISE assays with LUVs loaded with NaCl and suspended in media with various salts of different anions. The results obtained indicated that compound **1** (Figure 4) could mediate Cl^−^/NO_3_^−^ antiport most efficiently, followed by Cl^−^/HCO_3_^−^ antiport, while no response was observed in Na_2_SO_4_.

In 2009 appeared the first study focused on bicarbonate transport mediated by small molecules, where J. T. Davis, Gale, Quesada, and co‐workers studied the natural product prodigiosin **2** and the synthetic compounds **3 a**–**c** with a combination of ISE and ^13^C NMR assays (Figures 3 and 4).^[31]^ The four compounds studied showed clear transport activity, with prodigiosin **2** being around 100 times more efficient than 4,6‐dihydroxyisophthalamides **3 a**–**c**. With this work, the development of synthetic bicarbonate transporters gained significant interest, and it became a common practice to study the HCO_3_^−^ transport properties of compounds able to mediate Cl^−^/NO_3_^−^ antiport in certain research groups.

Various reports on artificial bicarbonate transporters appeared during the next two years (Figure 4). In 2010, three new studies appeared, describing the Cl^−^/HCO_3_^−^ antiport ability of tripodal (thio)ureas **4 a**–**d**,^[47]^ calix[4]pyrrole **5** ^[48]^ and ceramide **6**.^[39]^ The high concentrations of transporter required to study those compounds showed that their activity was much lower than that of prodigiosin **2**, but the observed transport properties were encouraging for the development of more efficient and structurally diverse chloride and bicarbonate transporters. In fact, it was only one year later when four new studies described various synthetic carriers with bicarbonate transport activities comparable to that of prodigiosin **2** (*see Section 3.5* for a detailed discussion on the most active bicarbonate transporters). The series of tripodal transporters **4** was extended with fluorinated tris(thio)ureas **4 e**–**l**, which showed up to 100 times increased Cl^−^/HCO_3_^−^ antiport activity.^[49]^ Monothioureas **7 a**–**c** were active as both Cl^−^/NO_3_^−^ and Cl^−^/HCO_3_^−^ antiporters, and **7 c** was significantly more active than the others.^[50]^ A family of *trans*‐decalin‐based bisureas was also reported as very efficient Cl^−^/NO_3_^−^ antiporters and the most active bisurea **8 a** showed also good Cl^−^/HCO_3_^−^ antiport activity.^[51]^ Moreover, the synthetic prodiginine obatoclax **9 a** and the structurally related compounds **9 b**–**e** were also studied as bicarbonate transporters.^[52]^

**Figure 5:**
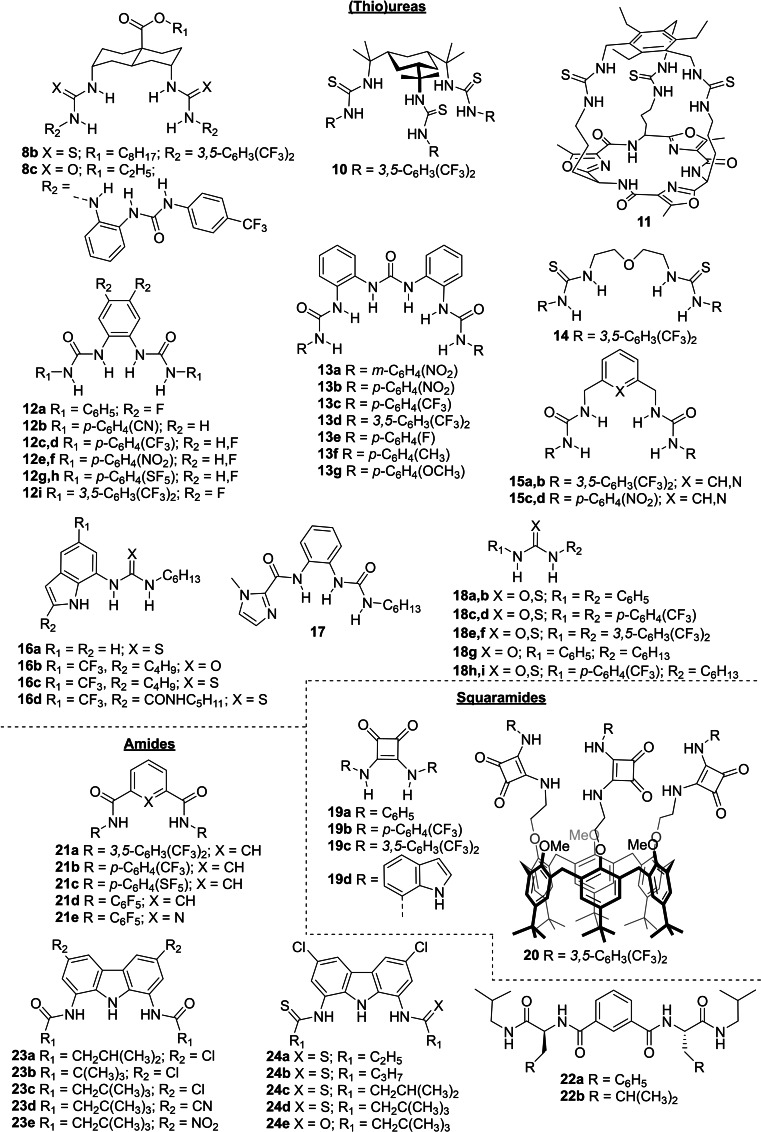
Small neutral molecules with acidic NH groups reported as HCO_3_^−^ transporters after 2011.

**Figure 6:**
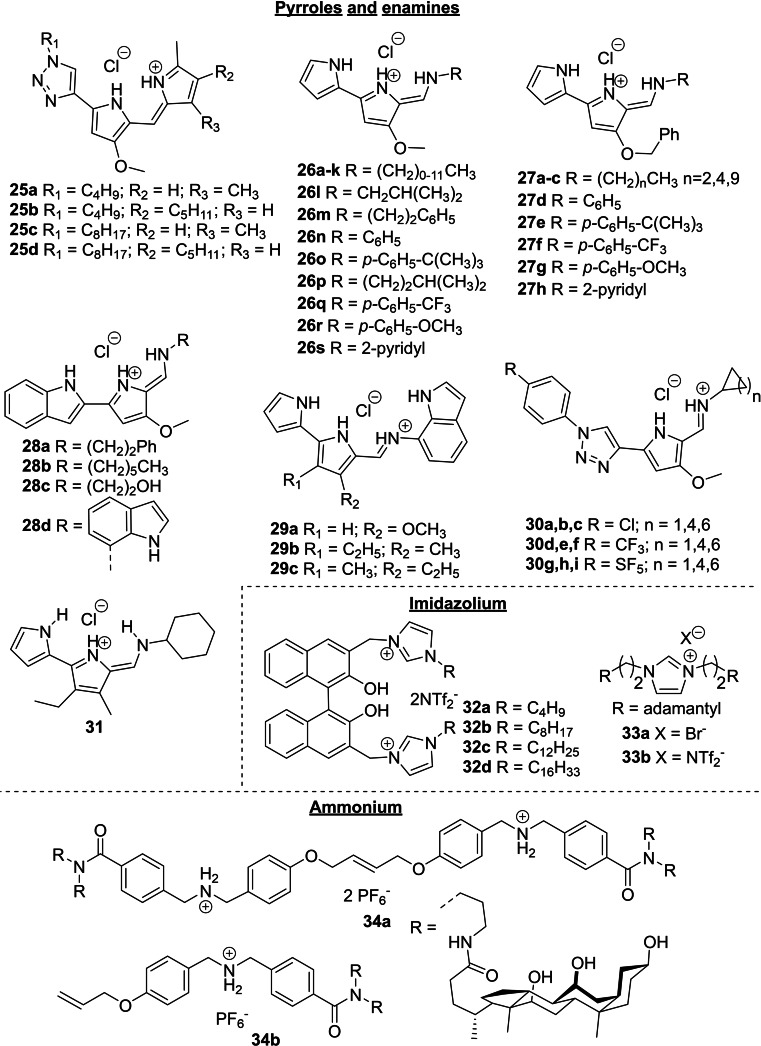
Cationic molecules reported as HCO_3_^−^ transporters after 2011.

**Figure 7:**
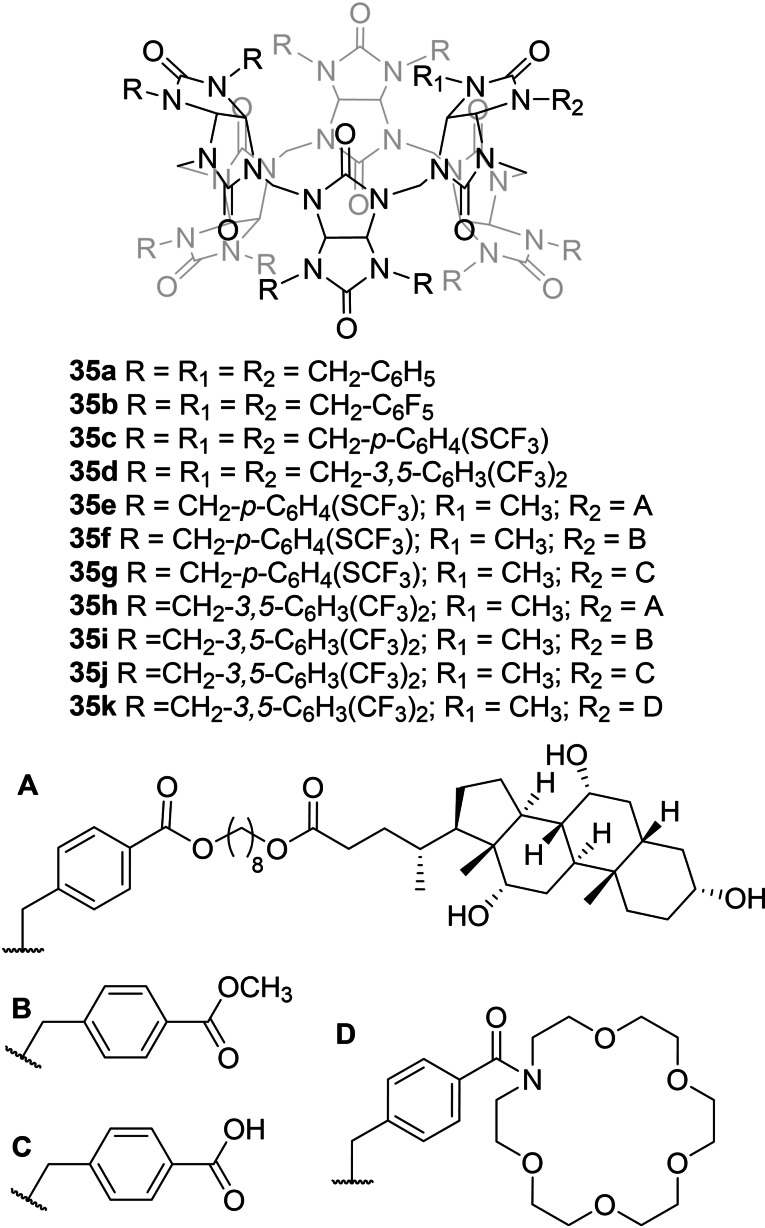
Bicarbonate transporters based exclusively on polarized CH bonds as the anion binding groups.

Often, anion transporters contain functional groups able to bind anions and extract them into the lipophilic interior of the bilayer membrane. The different bicarbonate transporters presented in this review are summarized in Figures 4, 5, 6, 7, where they are classified according to the type of anion binding groups present in their structures: (thio)ureas, squaramides, amides, pyrrole‐like rings, cationic groups, and polarized CH groups.

### 3.2 Neutral transporters with acidic NH groups

Ureas, thioureas, squaramides and amides are well known anion binding moieties with hydrogen bond donor properties coming from their acidic NH groups. Figures 4 and 5 show the different bicarbonate transporters based on those moieties, which can normally partition into the membrane as neutral molecules. The first works from A. P. Davis and Smith describing the ability of cholapods such as **1** to facilitate phospholipid flip‐flop and anion transport in membranes showed the potential of anion receptors in general, and of ureas in particular, as transmembrane anion transporters.[^30^, ^53^] Twenty years later, ureas and thioureas have become one of the most common functional groups used to develop mobile carriers for anion transport, including some of the most active transporters reported.[^41^, ^54^] The groups of A. P. Davis, Gale, Jolliffe, Valkenier, and Liu have reported a diverse collection of (thio)urea‐based bicarbonate transporters (Figure 4 and 5). Typically, this kind of transporters have structural cores able to pre‐organise the (thio)urea units for the efficient binding and consequent transport of the anions, as is the case of the steroid scaffold in the cholapods. The earliest examples of (thio)urea‐based bicarbonate transporters after the cholapods contained a tris(2‐aminoethyl)amine core, that yielded tripodal anion receptors **4 a**–**l**,[^47^, ^49^] and a *trans*‐decalin core, which is present in compound **8 a**–**c** and represents a structural simplification of the steroid scaffold in the cholapods.[^41^, ^45^, ^51^, ^55^] Other structural cores used to obtain tripodal transporters were the cyclohexane‐based scaffold in compound **10** and the tris(aminomethyl)benzene and cyclopeptidic scaffolds present in the capsular compound **11**.[^56^, ^57^] Moreover, various bis‐amine scaffolds were used to obtain (thio)ureas **12**–**15**.[^58^, ^59^, ^60^, ^61^, ^62^]

Although designing complex structures with multiple binding units is an interesting strategy to obtain efficient transporters, mono(thio)ureas **7 a**–**c** and **16**–**18** also showed to be active as Cl^−^ and HCO_3_^−^ carriers.[^50^, ^63^, ^64^, ^65^] The transport activities of these compounds indicate that aryl substituents afford more efficient (thio)urea transporters than alkyl substituents and that fluorination of those aryl substituents increases their transport efficiency even more, a general trend also observed in bis‐ and tris‐(thio)ureas. Moreover, the indole substituents in compounds **7 c** and **16 a**–**d** contribute to the binding and transport of the anion, yielding structurally simple compounds with interesting bicarbonate transport properties (*see Sections 3.5 and 4.1*).

Gale and co‐workers compared diaryl (thio)ureas **18 a**–**f** to analogous squaramides **19 a**–**c** and the squaramides showed higher activity than ureas and thioureas with the same aryl substituents, although the faster transport by squaramides was more noticeable for Cl^−^/NO_3_^−^ antiport (4–30 times faster) than for Cl^−^/HCO_3_^−^ antiport (∼2 times faster).^[65]^ Later, Caltagirone and co‐workers reported squaramide **19 d**, which contains indole substituents able to contribute to the binding of the anions.^[66]^ Although **19 d** is a better anion receptor than any of the squaramides **19 a**–**c**, it could only surpass the anion transport ability of squaramide **19 a**, but not that of the more active fluorinated derivatives **19 b**–**c**. In contrast to (thio)urea‐based transporters, bis‐ and tris‐squaramides have rarely shown better chloride transport activity than diaryl monosquaramides **19 b**–**c**,^[67]^ and the bicarbonate transport properties of such compounds have not been explored. Only in a recent study, Valkenier and co‐workers have described tris‐squaramide **20**, based on a calix[6]arene scaffold, as an efficient chloride and bicarbonate transporter.^[68]^

Isophthalamides **3 a**–**c** and ceramide **6** are early examples of amide‐based bicarbonate transporters. While the former contain two amide groups pre‐organized by an aryl scaffold to bind the same anion, the latter combines one amide and two alcohol groups as anion binding units.[^31^, ^39^] Isophthalamide and similar dipicolinamide groups were also included in the structure of transporters **21 a**–**e** and **22 a**–**b**.[^42^, ^69^] It should be noted that peptide‐derivatives **22 a**–**b** are among the few bicarbonate transporters that probably function as channels instead of as mobile carriers, as suggested by patch‐clamp experiments for **22 a**. Furthermore, Chmielewski and collaborators have reported the bicarbonate transport properties of compounds **23 a**–**e** and **24 a**–**e**, which consist of a carbazole scaffold functionalized with two amide or thioamide groups, respectively (see also Section 4.2).[^70^, ^71^, ^72^]

Like amides and ureas, pyrrole rings contain an acidic NH that makes them useful groups for the design of anion receptors and transporters. For example, prodigiosin **2** contains three interconnected pyrrole rings in its structure and the indole and carbazole scaffolds present in compounds **7 c**, **9 a**–**e**, **16 a**–**d**, **19 d**, **23 a**–**e**, and **24 a**–**e** are benzopyrrole rings that contribute to the binding and transport of the anions (Figures 4 and 5). Calix[4]pyrroles are tetrapyrrolic macrocycles whose HB donor properties have been extensively exploited.^[73]^ Although there are several examples of calix[4]pyrrole‐based anion transporters, only compound **5** has been reported as a bicarbonate transporter (Figure 4), while its non‐fluorinated analogue did not show significant Cl^−^/NO_3_^−^ or Cl^−^/HCO_3_^−^ antiport activity.^[48]^

### 3.3 Cationic transporters

Prodiginines and tambjamines are natural alkaloids with promising biological activities, including anticancer and antimicrobial properties. Quesada and co‐workers have reported a large variety of synthetic analogues of prodiginines and tambjamines, for which they have studied their anion transport properties in large unilamelar vesicles. These are then related to their biological activities studied in cells and tissues (*see Section 4.3 for an extended discussion*). Prodigiosin **2** is an example of a natural prodiginine and it contains a tripyrrole core, which is a common feature in this family of compounds. Thus, natural prodiginines and their synthetic analogues contain pyrrole rings in their structures as the principal anion binding groups. In compounds **9 a**–**e**, one of the pyrrole rings in natural prodiginines has been replaced by an indole ring (Figure 4).^[52]^ These compounds are readily protonated to afford cationic species with an additional NH that can contribute to the binding of the anion. The protonated receptor and the anion form a globally neutral complex, which can diffuse through the membrane as part of the transport process. In fact, this class of compounds is normally obtained and studied as their hydrochloric acid salts. Figure 6 shows other examples of bicarbonate transporters that probably mediate anion transport as cationic species. Compounds **25 a**–**d**, named click prodiginines, are also synthetic derivatives of prodigiosin **2** where one of the pyrrole rings was replaced by a triazole ring with a polarized CH able to coordinate anions.^[74]^

Compounds **26 b**, **26 k**–**m** and **26 p** are natural tambjamines and, as their synthetic analogues, their structures contain a pyrrolenamine scaffold. Natural and artificial tambjamines **26 a**–**s** and **27 a**–**h** permitted to study the effect of different aromatic and aliphatic substituents in the enamine and alkoxy moieties.[^75^, ^76^, ^77^] In fact, the study of tambjamines **26 a**–**k** and **27 a**–**c** was a pioneering work showing the role of lipophilicity in transmembrane anion transporters.^[76]^ Like in previous examples, the insertion of indole and triazole rings was also explored with this class of compounds. In compounds **28 a**–**d** one of the pyrrole groups of the 4‐methoxy‐2,2′‐bipyrrole core present in natural tambjamines was replaced by an indole ring, and in compounds **28 d** and **29 a**–**c** the indole was inserted as a substituent of the enamine group.[^78^, ^79^] Compounds **30 a**–**i** were named click tambjamines, due to the presence of a triazole ring that replaced one of the pyrrole rings in the natural predecessors. This permitted to explore structural changes by straightforward functionalization at both the triazole ring and the imine/enamine moiety.^[80]^ Moreover, in compound **31**, the typical methoxy and hydrogen substituents in the β positions of the central pyrrole ring were replaced by methyl and ethyl substituents respectively.[^81^, ^82^] This substitution pattern had already been used in compounds **29 b**–**c**, but while these compounds did not show remarkable activity product **31** is the most active tambjamine reported. The activity of this type of compounds was shown to be quite sensitive to subtle structural changes, and most of the designs explored afforded highly active transporters, but only with the right substituents (see Section 3.5 for an extended discussion on the most efficient transporters). In contrast to all the changes explored in the peripheral groups, a recent study shows that the central pyrrole ring is crucial for the transport activity of prodiginines and tambjamines, since its substitution by furan rings afforded poor transporters.^[83]^

Schmitzer and co‐workers have reported the cationic transporters **32**–**34**, which were studied as bromide, bistriflimide or hexafluorophosphate salts.[^84^, ^85^, ^86^] While compounds **32 a**–**d** and **33 a**–**b** contain cationic imidazolium rings, steroid‐based transporters **34 a**–**b** contain secondary ammonium groups. Based on the results from vesicles prepared with variable lipid compositions, the authors concluded that compounds **32 b**–**d** and **34 a**–**b**, with the largest structures, operated via a channel mechanism, while the smallest compounds worked exclusively as carriers. The results obtained suggested that all these compounds can transport bicarbonate, among other anions, with similar activities and selectivities (see Section 4.1 for further discussions).

### 3.4 Transporters without acidic NH or OH groups

Valkenier, Šindelář and co‐workers have reported bambusurils **35 a**–**d** as efficient Cl^−^/HCO_3_^−^ antiporters (Figure 7), showing also for this kind of transporters that fluorination of the aromatic substituents improved their transport properties.^[87]^ These macrocycles have a different anion binding nature to that of the NH‐based receptors summarized in previous sections. Bambusurils are neutral compounds that bear twelve polarized CH groups pointing to the interior of a cavity that can bind anions very efficiently. Monofunctionalised analogues of **35 c** and **35 d** were also prepared and conjugated to cholic acid and crown‐ether moieties (**35 e**–**k**).^[88]^ These substituents showed only a marginal effect on the transport properties of the bambusurils. Bambusurils represent one of the most active and selective families of bicarbonate transporters (see Sections 3.5 and 4.1 for extended discussions).

### 3.5 Overview of the most active transporters

**Figure 8:**
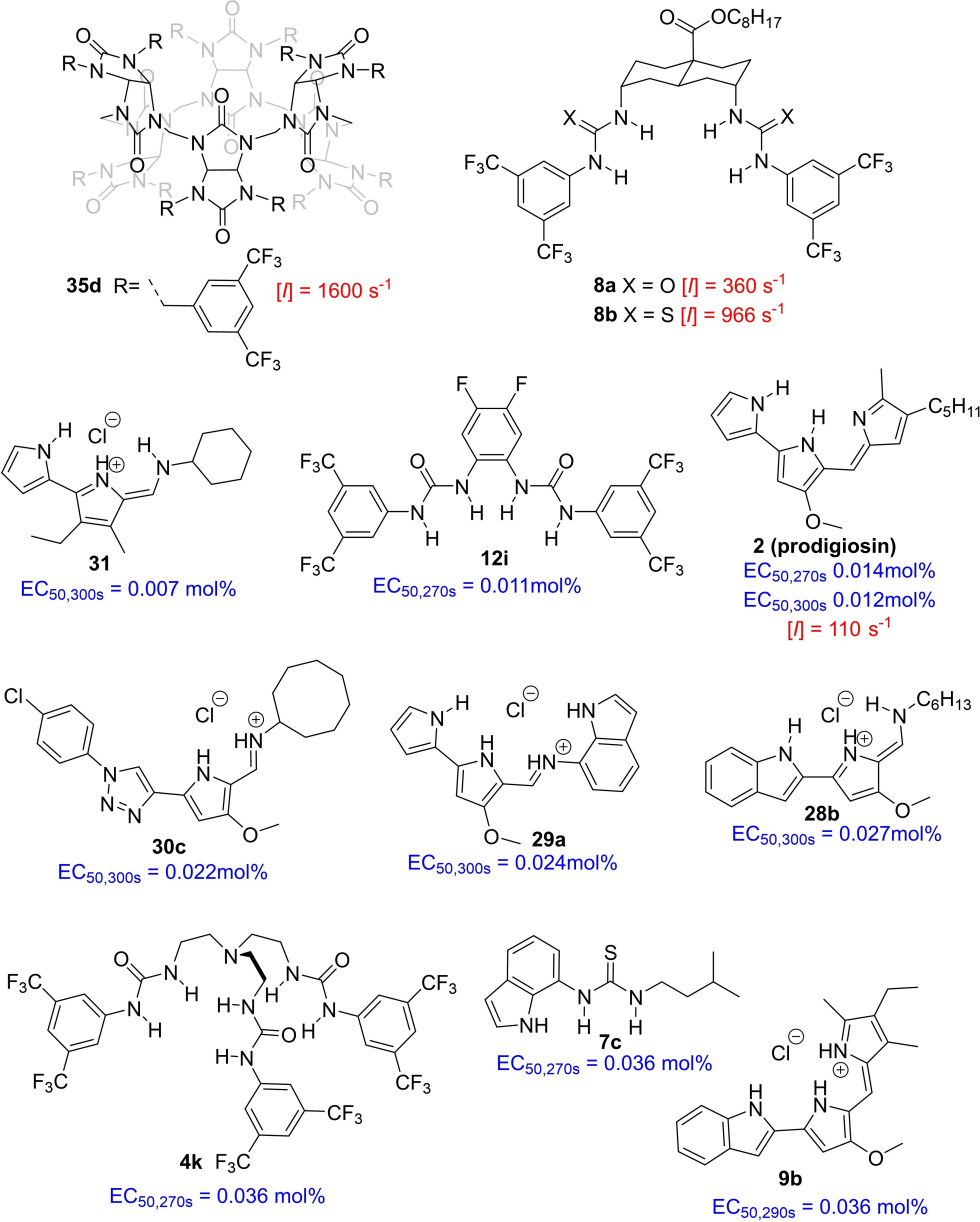
Most active bicarbonate transporters. EC_50_ values (in blue) indicate the concentration of transporter required for 50 % of the maximum effect in chloride selective electrode assays, using POPC LUVs. Specific initial rates [*I*] (in red) indicate the transport rates obtained from lucigenin assays, using 7 : 3 POPC‐cholesterol LUVs.

All the compounds described in the previous sections have shown activity in Cl^−^/HCO_3_^−^ antiport experiments, covering a wide range of activities. Figure 8 shows a selection of the most active transporters. Note that each compound in Figure 8 represents the most active transporter of a family of structurally related compounds, and in some cases, there are other compounds of remarkable activity in the same family.

Prodigiosin **2** was the first compound reported as a highly efficient bicarbonate transporter, because clear transport was observed in a chloride selective electrode assay at a concentration as low as 0.05 transporters per 100 lipid molecules (0.05 mol %).^[31]^ In later studies, this compound has been used as a reference of high Cl^−^/HCO_3_^−^ antiport activity, being studied in most of the assays discussed in Section 2.

Since EC_50_ values represent the concentration of transporter required to obtain a 50 % chloride efflux at a concrete time of transport in the ISE assay, the lower the EC_50_ the higher the activity. The EC_50_ value determined for prodigiosin **2** was 0.014 mol %^[59]^ and that obtained with compounds **4 k**, **7 c** and **9 b** was 0.036 mol % in all three cases,[^49^, ^50^, ^52^] indicating that prodigiosin **2** is only around 3 times more active than these compounds. *Ortho*‐phenylene bis‐ureas **12 d**, **12 h** and **12 i** were slightly more efficient than prodigiosin, with EC_50_ values of 0.011–0.012 mol % (Figures 4 and 8).^[59]^ The families of tambjamines **26**–**31** described before were also studied with ion selective electrode assays. Compounds **26 f**, **26 g**, **27 b**, **28 b**, **29 a**, **30 b**–**c** and **30 e**–**f** stand out from the rest, with EC_50_ values in the range of 0.022‐0.029 mol %,[^76^, ^78^, ^79^, ^80^] which is about half the activity of prodigiosin (Figures 6 and 8).^[74]^ Moreover, tambjamine **31** was recently found to have an unprecedented EC_50_ value of 0.007 mol %,^[82]^ outperforming prodigiosin **2**. It should be noted that the times at which the EC_50_ values have been determined vary between 270 and 300s (indicated as a subscript in Figure 8), which can lead to subtle differences in the calculated values, as observed for prodigiosin **2** (0.014‐0.012 mol %). Thus, accurate comparison between close values determined at different times is not reliable (e. g., between compounds **31** and **12 i**).

It is worth to note that the family of tripodal (thio)ureas **4 a**–**l** was first studied in 2011 with the commonly used ISE assay (with LUVs suspended in Na_2_SO_4_).^[49]^ In 2018 these compounds were reinvestigated with the osmotic assay and with a modified ISE assay (with LUVs suspended in Na‐gluconate).^[44]^ These two new methods avoided any background response that could be caused by transport of sulphate species and showed a trend in activity which was different to that observed in the classical ISE assay, with **4 h** as the best bicarbonate transporter of the series (Figure 4). Although **4 k** had been previously reported as the most efficient bicarbonate transporter, this compound showed a poorer activity in the new assays. This is an unexpected result that could not be explained just based on the interference from sulphate transport, but which might be caused, for example, by differences in deliverability of the transporters in different salt solutions. This contrast in the results obtained from the different experiments reveals the importance of combining various assays to afford a deeper understanding of the transport process.

The transport curves from a lucigenin assay can be used to obtain the specific initial rate [*I*], which is indicative of the activity of the transporter at the beginning of the experiment, independent of the concentration. Decalin bis‐urea **8 a** showed good Cl^−^/HCO_3_^−^ antiport activity in the lucigenin assay at 0.004 mol %, and the [*I*] determined for this compound was 360 s^−1^.[^45^, ^51^] The analogous bis‐thiourea **8 b**, one of the most efficient chloride transporters described to date,^[41]^ showed to be more active for both Cl^−^/NO_3_^−^ and Cl^−^/HCO_3_^−^ antiport, with a [*I*] of 970 s^−1^ for the latter process.^[45]^ Prodigiosin **2** also was studied with the lucigenin assay, giving a [*I*] of 110 s^−1^, which revealed that decalins **8 a**–**b** are among the few synthetic transporters with significantly higher efficiency than prodigiosin **2**.^[87]^ Moreover, bambusuril **35 d** afforded a specific rate [*I*] of 1600 s^−1^.^[87]^ This compound has shown the highest activity in the lucigenin assay, showing efficient transport even at 0.001 mol %. It is impossible to directly compare data from different assays in a quantitative way, but the fact that bambusuril **35 d** showed a specific rate more than ten‐fold higher than prodigiosin **2** in the lucigenin assay, while **31** and **12 i** performed only marginally better than **2** in the ISE assay, supports the conclusion that bambusuril **35 d** and its analogues (such as **35 h** and **35 k**) are the most active HCO_3_^−^ transporters reported to date.

Compounds **2**, **8 a**–**b** and **35 d**, the most active compounds tested in the lucigenin assay, were also studied with the EuL1 assay.^[45]^ This assay confirmed the high bicarbonate transport efficiency of these compounds, which all showed clear activity at 0.004 mol %. Although with this method all the transporters showed similar activity, mechanistic studies revealed that bambusuril **35 d** was clearly the most active transporter via a classical HCO_3_^−^/Cl^−^ antiport mechanism (see Section 4.2 for a detailed discussion on mechanistic studies). Moreover, this compound also showed the best activity in HCO_3_^−^ uniport experiments and performed in a follow‐up study with more bambusurils still the best, together with **35 k**.^[88]^ The special character of the bambusuril binding site (formed by polarized CH groups), the high anion affinity, the large degree of fluorination, and the possibility to accommodate two anions in its binding site are features that probably contribute to the exceptional transport properties of these fluorinated bambusurils.

## 4 Further discussions and developments

### 4.1 Anion transport selectivity

The chloride selective electrode and the lucigenin assays have been commonly used to study Cl^−^/HCO_3_^−^, Cl^−^/NO_3_^−^, and Cl^−^/SO_4_^2−^ antiport processes, because such processes are monitored by direct detection of chloride either outside or inside LUVs (see Section 2 for details on the assays). The three anions of which antiport with chloride is commonly studied have different hydration energies, in the order of SO_4_^2−^ (−1080 kJ mol^−1^) > HCO_3_^−^ (−335 kJ mol^−1^) > NO_3_^−^ (−300 kJ mol^−1^).^[89]^ Thus, SO_4_^2−^, a bivalent anion and with the highest hydration energy, is very difficult to extract into the lipophilic interior of the membrane, and most mobile carriers cannot perform Cl^−^/SO_4_^2−^ antiport. The hydration energy for Cl^−^ (−340 kJ mol^−1^) is similar to that for HCO_3_^−^, and based on this, we would expect that HCO_3_^−^ could be transported at rates similar to Cl^−^, while NO_3_^−^ would be easier to transport. However, recent publications have shown that trends in selectivity for anion transport are more complex.^[90]^

The general trend with the ion selective electrode assays is that EC_50_ values obtained for Cl^−^/HCO_3_^−^ antiport are around one order of magnitude higher than those for Cl^−^/NO_3_^−^ antiport, which would agree with faster transport of NO_3_^−^ compared to that of HCO_3_^−^. However, in the ISE assay the extravesicular concentration of NO_3_^−^ is typically 10‐fold higher than that of HCO_3_^−^, which could also contribute to the differences in EC_50_ values normally observed. To obtain a fairer comparison of the two processes, analogous conditions should be used in the two assays.[^63^, ^64^, ^91^] Nevertheless, deviations from that general trend have been observed and some compounds, such as **18 e**–**f** and **7 b**–**c**,[^50^, ^65^] gave similar EC_50_ values for Cl^−^/NO_3_^−^ and Cl^−^/HCO_3_^−^ transport, which could indicate that Cl^−^ transport is rate limiting for those compounds.[^90^, ^92^]

In the lucigenin assay, the concentration of the anion coupled to Cl^−^ is normally the same, and therefore the transport curves can be used to compare the efficiency of the transporters to perform Cl^−^/NO_3_^−^ and Cl^−^/HCO_3_^−^ antiport.^[93]^ In general, transporters perform Cl^−^/NO_3_^−^ faster than Cl^−^/HCO_3_^−^ antiport, as has been observed for compounds **1**, **6**, **8 b**, **10** and **14**. On the other hand, ureas **8 a** and **8 c** showed similar transport efficiency for both antiport processes, despite their structural similarity to thiourea **8 b**. Cationic transporter **32 b** and **33 b** showed slightly faster Cl^−^/HCO_3_^−^ than Cl^−^/NO_3_^−^ antiport and transport in presence of SO_4_^2−^ was found to be even faster. However, these trends could originate from the high concentrations used (5–15 mol %) and the impact of the counter anion on their deliverability.

In contrast to the general behaviour observed for anion carriers, bambusurils **35 c**–**d** have shown an unprecedented preference for Cl^−^/HCO_3_^−^ antiport, which was more than 100 times faster than Cl^−^/NO_3_^−^ antiport.^[87]^ Moreover, compound **35 d** also performs HCO_3_^−^/Cl^−^ antiport more than 40 times better than HCO_3_^−^/NO_3_^−^ antiport.^[45]^ The main reason for the poor transport activities of these macrocycles in presence of NO_3_^−^ is the formation of very stable complexes between NO_3_^−^ and the bambusuril in the membrane, limiting the release of the anion and resulting in the blocking of the binding site. In contrast, (thio)ureas **8 a**–**b** and prodigiosin **2** showed similar activities for HCO_3_^−^/Cl^−^ and HCO_3_^−^/NO_3_^−^ antiport.

**Figure 9:**
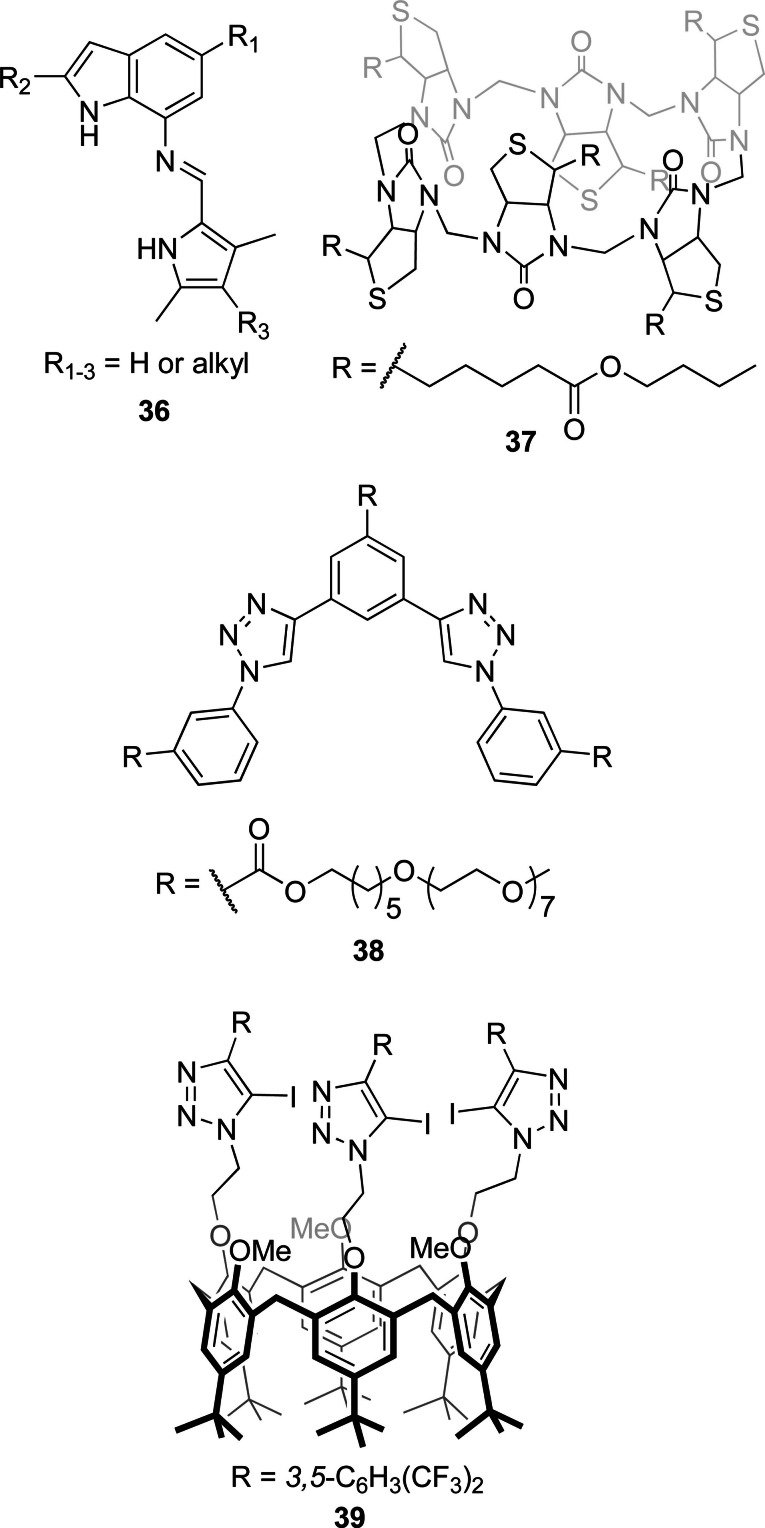
Efficient Cl^−^ transporters that showed no activity as HCO_3_^−^ transporters.

Although transport of bicarbonate is in general more challenging than transport of nitrate, most transporters able to mediate Cl^−^/NO_3_^−^ antiport efficiently and tested for Cl^−^/HCO_3_^−^ transport, did show at least some activity. There are only a few cases of efficient Cl^−^/NO_3_^−^ antiporters that have not shown any activity in a Cl^−^/HCO_3_^−^ antiport assays. The structures of those compounds are shown in Figure 9. Perenosins **36** are imine‐based compounds that rely on protonation to transport anions, and their inability to transport bicarbonate has been explained by the deprotonation of the active species caused by the basic nature of bicarbonate.^[94]^ Biotinuril **37** and bis(aryl‐triazole) **38** are anion receptors based on polarized CH groups that also showed remarkable selectivity for Cl^−^/NO_3_^−^ over Cl^−^/HCO_3_^−^.[^40^, ^95^] These compounds bind anions via a multiple C−H⋅⋅⋅anion interactions and a preference for soft anions (such as Cl^−^) over harder anions (HCO_3_^−^) was suggested to be the reason for such selectivity. However, this is not a general feature of receptors with polarized CH groups, as shown by the high activity of bambusurils **35 a**–**d**. The halogen bond‐based transporter **39** contains a calix[6]arene scaffold similar to that of squaramide **20**. While these two compounds performed Cl^−^/NO_3_^−^ antiport with similar activities, **39** is inactive as Cl^−^/HCO_3_^−^ antiporter under the conditions used to obtain efficient transport by **20**.^[68]^ It should be noted that most of these transporters with remarkable selectivity for Cl^−^/NO_3_^−^ antiport have no acidic NH or OH groups. We suggest that the poor response obtained for these compounds in Cl^−^/HCO_3_^−^ antiport assays might be related to their inability to perform H^+^Cl^−^ symport, a process that can lead to net Cl^−^/HCO_3_^−^ antiport, as discussed in Section 4.2.

### 4.2 Mechanistic studies

**Figure 10:**
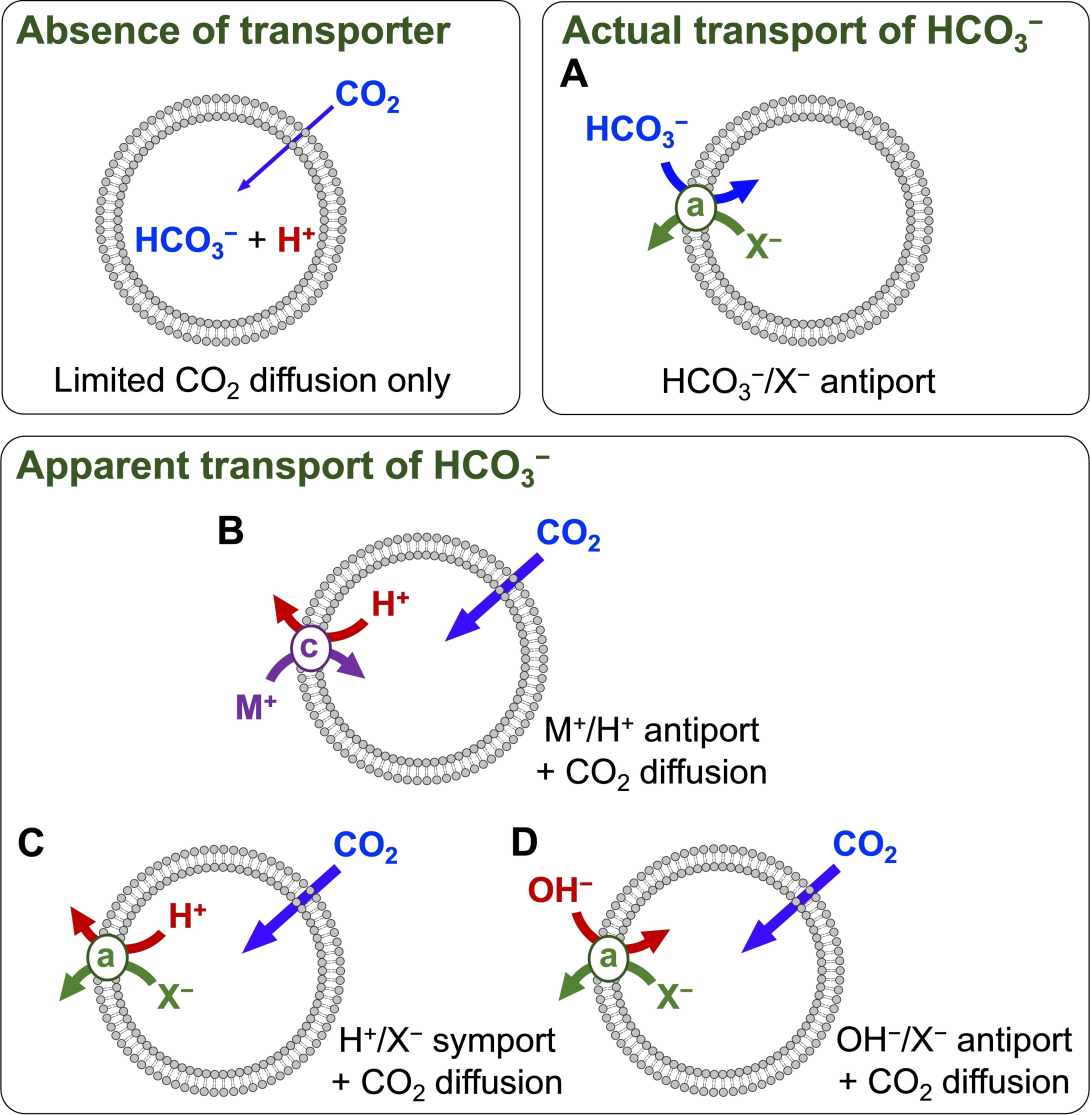
Different mechanisms by which net transport of HCO_3_^−^ could occur in presence of a concentration gradient of this anion. In absence of a transporter, only a small amount of CO_2_ will diffuse across the membrane, leading to an acidification of the interior of the LUVs. In mechanism A, an anionophore (a) exchanges HCO_3_^−^ for another anion. Mechanisms B−D rely on the diffusion of CO_2_ coupled to transport of H^+^ or OH^−^ by cationophores (c) or anionophores to result in the net transport of HCO_3_^−^, without this anion crossing the membrane.

In biological processes, the transport of bicarbonate is often linked to the diffusion of CO_2_, as elaborated on in Section 1.1 of this review. Using the newly developed EuL1 assay, we have recently shown that in addition to *actual* transport of HCO_3_^−^, CO_2_ diffusion can also lead to *apparent* transport of HCO_3_^−^ into LUVs^[45]^ (Figure 10) and Gale and co‐workers have also considered alternative mechanisms of bicarbonate transport in a recent publication.^[44]^

Upon addition of HCO_3_^−^ to LUVs in absence of any transporter, some CO_2_ can diffuse into the LUVs, where it will react with H_2_O into HCO_3_^−^ and H^+^ (Figure 10) leading to an acidification of the interior, as demonstrated with the pH‐sensitive probe HPTS.^[45]^ The diffusion of CO_2_ will stop when the pH gradient across the liposome membrane reaches an equilibrium with the HCO_3_^−^ gradient in the opposite direction, or when the CO_2_ concentration inside the LUVs (at pH<7) is identical to that exterior (at pH∼7.4). In the EuL1 assay, this process leads to a minimal amount of HCO_3_^−^ entering the LUVs when using a buffer solution with 5 mM HEPES at pH 7. A higher buffer concentration (20 mM, pH 7.6) led to more HCO_3_^−^ inside the LUVs, as the buffer prevented the acidification of the interior of the LUVs upon diffusion of CO_2_.^[45]^

In LUVs containing a HCO_3_^−^/Cl^−^ antiporter, the addition of HCO_3_^−^ triggers the HCO_3_^−^/Cl^−^ exchange process, which equilibrates the concentrations of HCO_3_^−^ inside and outside the vesicles (Figure 10, A). The concentrations of CO_2_ and the pH are linked to the concentration of HCO_3_^−^ (see Scheme 1) and will thus be equilibrated as well.

In the presence of any transporter that can dissipate the pH gradient caused by the diffusion of CO_2_ into the LUVs, the diffusion can continue until the concentrations of both CO_2_ and HCO_3_^−^ (and the pH) are identical in the interior and exterior of the LUVs. This could be achieved with a cation transporter such as monensin, which is able to efficiently perform H^+^/Na^+^ or H^+^/K^+^ transport, but also by anionophores that can perform H^+^X^−^ symport or OH^−^/X^−^ antiport (Figure 10, B−D). All these processes will result in *apparent* transport of HCO_3_^−^, as the concentration of HCO_3_^−^ inside the LUVs will increase significantly, without any HCO_3_^−^ anions actually crossing the membrane.

Most assays for HCO_3_^−^ transport cannot distinguish between these mechanisms. For instance, in the ISE assay, a pulse of NaHCO_3_ is added to the LUVs, leading to a CO_2_ gradient across the membrane, while a Cl^−^ gradient is also present. Thus, diffusion of CO_2_ into the LUVs facilitates HCl efflux, which would in absence of HCO_3_^−^ be limited by the build‐up of pH gradient (basification of the interior). In the lucigenin assay, no gradients of HCO_3_^−^ or CO_2_ are present, but the high NaHCO_3_ concentration in the experiments (225 mM) can serve as a buffer, thus allowing HCl influx into the LUVs. The ^13^C NMR assay reveals interior HCO_3_^−^ and does also give a response upon *apparent* HCO_3_^−^ transport, as demonstrated with monensin.^[45]^

In contrast, the EuL1 assay can distinguish between *actual* and *apparent* transport of HCO_3_^−^.[^45^, ^72^] The rate of *apparent* HCO_3_^−^ transport is often limited by the diffusion of CO_2_, provided that the transporter is dissipating the pH gradient sufficiently fast, as is the case when monensin is added at a concentration of 0.1 mol %. If an anion transporter operates primarily via mechanism C or D and CO_2_ diffusion is rate limiting, then the addition of monensin will not change the overall rate of HCO_3_^−^ transport, as this will remain limited by CO_2_ diffusion. This was the case for anion transporters **2**, **8 a**, **8 b**, **23 c**–**e**, and **24 d**. However, if an anion transporter operates primarily via the *actual* HCO_3_^−^ transport mechanism A, addition of monensin adds an additional transport pathway (B) and the overall rate of transport will increase. This was observed for bambusuril **35 d**. We note that the main mechanism of transport cannot be determined by addition of monensin if a transporter gives only low rates of overall HCO_3_^−^ transport, which would be negligible compared to the transport by monensin via mechanism B. On the other hand, when the transport rates clearly surpass the CO_2_ diffusion limited rate, then we can conclude that *actual* HCO_3_^−^ transport (mechanism A) must take place. This was observed for decalins **8 a**–**b**,^[45]^ and also for carbazoles **23 d**–**e** and **24 d**.^[72]^

**Figure 11:**
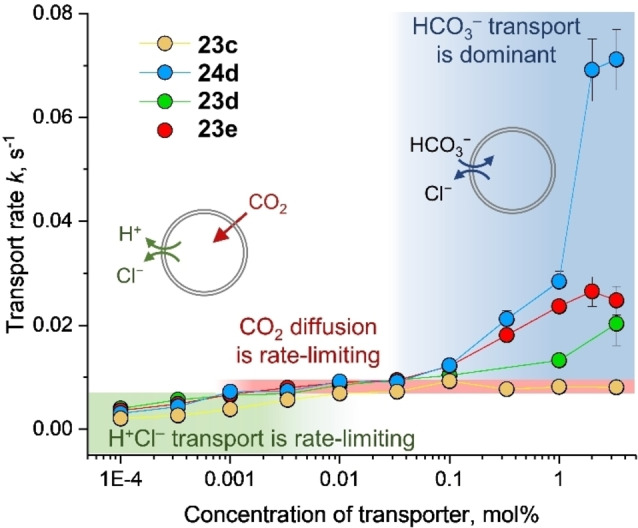
Rate constants as measured in the EuL1 assay for HCO_3_^−^ transport for different concentrations of carbazole‐based transporters, showing that different mechanisms dominate at different stages. Reproduced from Ref. [72] with permission from the Royal Society of Chemistry.

Compounds **23 c**–**e** and **24 d** were studied at a range of concentrations spanning 5 orders of magnitude and rate constants were determined (Figure 11).^[72]^ At very low concentrations (<0.001 mol %), the transport of H^+^Cl^−^ (or equivalent OH^−^/Cl^−[96]^) is rate limiting, and the rate of net influx of HCO_3_^−^ increases with the concentration of transporter. From 0.001 mol % (**23 d**–**e** and **24 d**) or 0.01 mol % (**23 c**), the global rate of HCO_3_^−^ transport is nearly independent of the transporter concentration over several orders of magnitude of concentration. This plateau in the transport rates corresponds to the CO_2_ diffusion limited rate as observed for monensin. At higher concentrations of **23 d**–**e** and **24 d** (>0.1 mol %), the rate of HCO_3_^−^ transport increases again, but in this case due to *actual* HCO_3_^−^/Cl^−^ exchange, and the rate constants for this mechanism (A) was determined by subtracting the rate constant for the CO_2_ diffusion limited transport from the total rate constants.^[72]^ Of the tested carbazoles, the thioamide **24 d** showed the highest rates of *actual* Cl^−^/HCO_3_^−^ transport, albeit its activity was ∼75‐fold lower than bambusuril **35 d**.

These mechanistic insights explain why most of the anion transporters that have shown activity for transport of Cl^−^ and NO_3_^−^ did also show activity for Cl^−^/HCO_3_^−^ antiport. Many of the compounds described as HCO_3_^−^ transporters have acidic NH groups (Figures 4 and 5) that could potentially deprotonate upon release of Cl^−^, resulting in a net transport of HCl. Other compounds were shown to be active in their protonated form (Figure 6) and these were also found to be efficient transporters of HCl in HPTS‐based methods. Thus, many of these HCO_3_^−^ transporters are likely to act via transport mechanism C (or D), as exemplified by transporters **2**, **8 a**, **8 b**, **23 c**–**e**, and **24 d**.[^45^, ^72^] On the other hand, most of the anion transporters that showed no activity for HCO_3_^−^ transport (Figure 9) do not have acidic H‐bond donor groups and are thus unlikely to transport HCl via deprotonation. Furthermore, they have softer H‐bond or halogen bond donor groups that are less likely to bind the hard OH^−^ anion. This eliminates the transport via mechanisms C and D by these compounds. In contrast to bambusurils **35**, they are not active via mechanism A either.

### 4.3 Bicarbonate transporters in cells and tissues

The biological effects of several of the anion transporters described in Section 3 of this review have been evaluated. Certain series of compounds, such as (thio)ureas **4** and **12** and tambjamine analogues **9** and **26**–**28**, showed clear cytotoxicity,[^49^, ^52^, ^58^, ^75^, ^77^, ^78^, ^80^, ^97^] and some tambjamine analogues and carbazoles showed antimicrobial activity as well.[^72^, ^79^] The toxicity correlated in many cases with anion transport activity and a clear perturbation of the pH of certain organelles was observed, as well as cell death by apoptosis. Thus, HCl transport is likely to be the main cause of the observed cytotoxicity in most of these cases, which could lead to applications of these compounds as anti‐cancer or antibiotic agents.

The deficient Cl^−^ and HCO_3_^−^ transport in the disease CF (see Section 1.1) has motivated anion transport studies in epithelial cell lines, using electrophysiological studies[^42^, ^98^, ^99^] or by monitoring the quenching of yellow fluorescent protein (YFP) by iodide entering the cells via I^−^/Cl^−^ antiport.^[99]^ A wide range of Cl^−^ transporters has been tested by the YFP method and showed encouraging anion transport activity.[^74^, ^80^, ^91^, ^100^, ^101^] Prodigiosin (**2**), Obatoclax (**9 a**), and compounds **25 c** and **31** were also found to cause an increase of the intracellular pH upon perfusion of cells with a solution of NaHCO_3_ (after a pre‐treatment with NH_4_Cl), indicating HCO_3_^−^ transport into the cells.^[81]^

**Figure 12:**
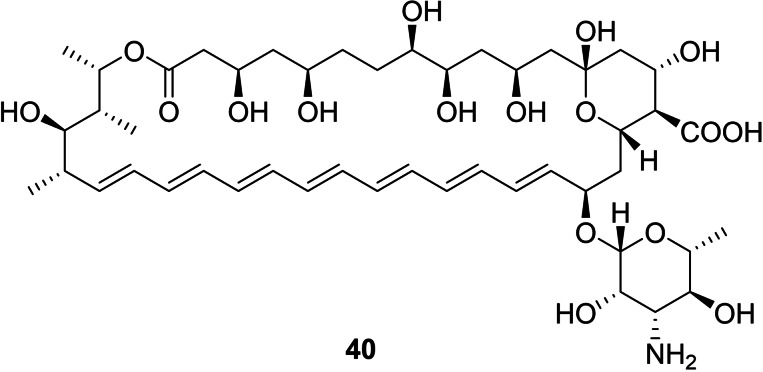
Natural HCO_3_^−^ transporter Amphotericin B.

The disease CF has a drastic impact on the properties of airway surface liquid (ASL), originating from the impaired transport of HCO_3_^−^ and Cl^−^ and resulting in ‘sticky mucus’.^[102]^ Encouragingly, the treatment of CF epithelial tissue with synthetic tambjamines **30 b** and **31** was shown to increase the pH and the volume of the ASL, while decreasing the viscosity.^[82]^ Similar results were obtained with the channel forming natural product amphotericin B (**40**, Figure 12), which was shown to transport not only cations and Cl^−^, but also HCO_3_^−^.^[43]^ Additionally, this study showed less bacterial activity in the ASL of cultured epithelia and improvements in the ASL of CF pigs in vivo. These highly promising results were attributed primarily to the transport of HCO_3_^−^ by **40**.

## 5 Conclusions

In this Review we have given an overview of the different synthetic anion receptors that were demonstrated to be active as bicarbonate transporters. The majority of these compounds uses ureas, thioureas, amides, thioamides or pyrrolic groups to interact with anions via NH‐based H‐bond donor groups. These compounds can typically perform Cl^−^/NO_3_^−^ and Cl^−^/HCO_3_^−^ antiport, but also H^+^Cl^−^ symport (or OH^−^/Cl^−^ antiport) and have thus a limited selectivity. Recent studies have shown that the ability of these compounds to dissipate pH gradients could lead to *apparent* HCO_3_^−^ transport, a process that relies on CO_2_ diffusion rather than on transport of HCO_3_^−^ anions across the membrane. Efficient anion transporters, such as decalin bisurea **8 a** and bisthiourea **8 b** and carbazole bisamides **23 c**–**e** and bisthioamide **24 d**, were found to act via the CO_2_ diffusion mechanism when present at low transporter concentrations, while true HCO_3_^−^/Cl^−^ antiport is the main mechanism at higher concentrations. To the best of our knowledge, bambusurils (**35**) are the only synthetic transporters that preferentially act via HCO_3_^−^/Cl^−^ antiport and that achieve high rates of transport. However, the fluorinated bambusurils are too lipophilic to be deliverable and to have any biological activity. For therapeutic applications, it would thus be of great interest to develop true HCO_3_^−^ transporters without acidic NH groups, that could be tested on cells and tissues. On the other hand, compounds such as synthetic tambjamines **30 b**, **31** and Amphotericin B **40** are not selective HCO_3_^−^ transporters; nevertheless, they have shown very promising results on cells, tissues and even in animal studies. Apart from biological applications in the context of *cystic fibrosis* or other channelopathies, HCO_3_^−^ transporters could also find applications in sensing and CO_2_ concentration. Furthermore, the lessons learned from all these studies on synthetic HCO_3_^−^ transporters have also deepened the fundamental insights in ion transport processes by ionophores in general and demonstrated how compound structures can be optimised to achieve maximal activities.

## Conflict of interest

The authors declare no conflict of interest.

6

## Biographical Information

*Luis Martínez‐Crespo was born in Felanitx (Spain) in 1985. He received his MSc in 2011 and his PhD in 2015 from the University of the Balearic Islands (UIB), under the supervision of Prof. Antonio Costa and Dr. Carmen Rotger. He has worked as a post‐doctoral researcher with Prof. Pablo Ballester in the Institute of Chemical Research of Catalonia (ICIQ), with Dr. Hennie Valkenier at the Université Libre de Bruxelles (ULB) and with Prof. Simon Webb at the University of Manchester (UoM). Currently, he works as a Maria Zambrano fellow at the UIB, with Prof. Antonio Costa. His research focuses on the development of supramolecular systems for transmembrane transport and signalling*.

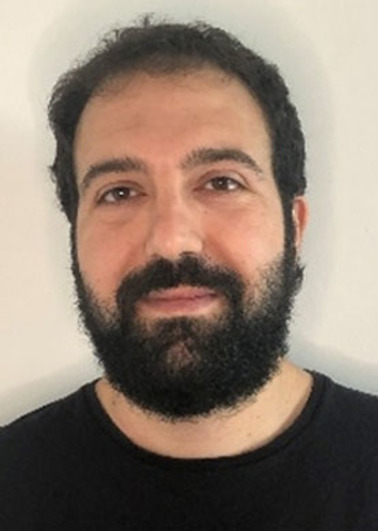

## Biographical Information

*Hennie Valkenier is originally from The Netherlands, where she studied at the University of Groningen (MSc in 2006, PhD in 2011). She has worked as a post‐doc with Prof. Anthony Davis at the University of Bristol and with Profs. Kristin Bartik and Gilles Bruylants at the Université libre de Bruxelles (ULB), before getting a permanent position as FNRS researcher in the Engineering of Molecular NanoSystems laboratory at the ULB in 2018. She was awarded an ERC starting grant that same year. With her team and collaborators, she is developing synthetic transporters for ions and new methods to study transport processes in liposomes*.

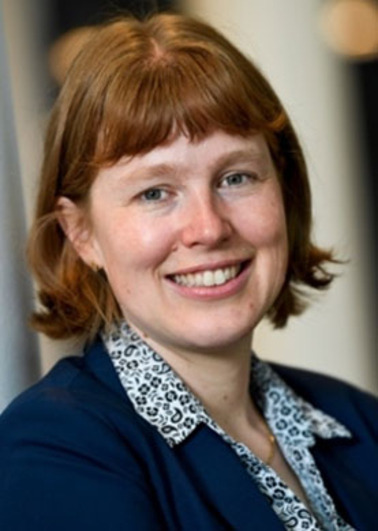

## Data Availability

Data sharing is not applicable to this article as no new data were created or analyzed in this study.
